# National Estimates of Human Flourishing and Associated Acts of Charity

**DOI:** 10.2196/90951

**Published:** 2026-07-23

**Authors:** Moshe Shegal, Peter C Austin, Eyal Cohen, Jennifer A Jairam, Jennifer S Wortham, Timothy Lomas, Joel G Ray

**Affiliations:** 1 St. Michael's Hospital, 30 Bond St, Toronto, ON, M5B 1W8, Canada, 1 416-864-6060; 2 Institute for Clinical Evaluative Sciences, Toronto, ON, Canada; 3 University of Toronto, Toronto, ON, Canada; 4 Harvard University, Cambridge, MA, United States

**Keywords:** charitable giving, volunteering, human flourishing, donations, social programs

## Abstract

Higher levels of human flourishing were moderately associated with helping strangers and volunteering time but showed little association with financial donations.

## Introduction

Charitable giving can be both fiscal and nonfiscal. Prosocial “acts of charity” can improve the health of both the donor and the recipient [1]. Charitable giving varies by country, as demonstrated by the Charity Aid Foundation’s World Giving Index (WGI) [2]. However, national predictors of charitable giving are understudied, including whether they are financial or nonfinancial in nature.

The Global Flourishing Study’s (GFS’s) Flourishing Index (FI) is a composite index comprising two self-report questions in five domains: happiness, health, meaning, character, and relationships [3]. Responses were aggregated into domain-specific scores and combined into an overall index. The mean scores were weighed to be nationally representative within each country. Accordingly, the current study evaluated the relation between the GFS’s FI and the WGI’s nation-based indicators of charitable giving.

Several of the domains that comprise the FI are theoretically linked to prosocial behavior. However, monetary forms of charity may rely on distinct factors. Therefore, this study examined whether human flourishing is associated with monetary and nonmonetary forms of charitable giving.

This study contributes to the current understanding of prosocial behavior by incorporating the multidimensional measure of human flourishing. Instead of considering charitable behavior as the sole outcome of economic indicators, this study evaluates how various domains of well-being are associated with civic engagement.

The salutogenic model emphasizes the social determinants of health that promote well-being, many of which are reflected in both the FI and the WGI. These social factors play a key role in public health. For example, stronger social cohesion may increase the tendency for individuals to engage in charitable giving while also supporting healthier behaviors. In one Dutch study, the salutogenic model was used effectively to create an intervention for healthy eating among patients with type 2 diabetes [4].

## Methods

### Overview

The FI for 22 countries was ascertained within Wave One of the GFS from 2022 to 2023, comprising more than 200,000 representatively sampled individuals. Country-level FI responses are scored on a continuous scale from 0 to 10. A higher score indicates greater flourishing. To ensure cross-country comparability, identical survey questions and response scales were used. Survey translation accuracy was confirmed by scholars [3].

The 2022 WGI examined national charitability and asked residents of a given country whether they have done any of the following within the past month: (1) helped a stranger who needed help, (2) donated money to charity, and (3) volunteered their time to an organization [2]. Each of the three WGI nation-level summary scores were plotted in relation to the FI, and the correlation expressed as an *R*^2^ and 95% CI.

Given the limited number of country-level observations (n=22), the results of this analysis are intended to illustrate descriptive patterns. The analysis does not adjust for income per capita, inequality, and other macro covariates. Therefore, these results should be interpreted as exploratory rather than causal.

### Ethical Considerations

In accordance with the Tri-Council Policy Statement, this study was exempt from a formal Research Ethics Board (REB) review. As per article 2.2, REB review is not required for research that relies exclusively on information in the public domain.

## Results

**Figure 1.**
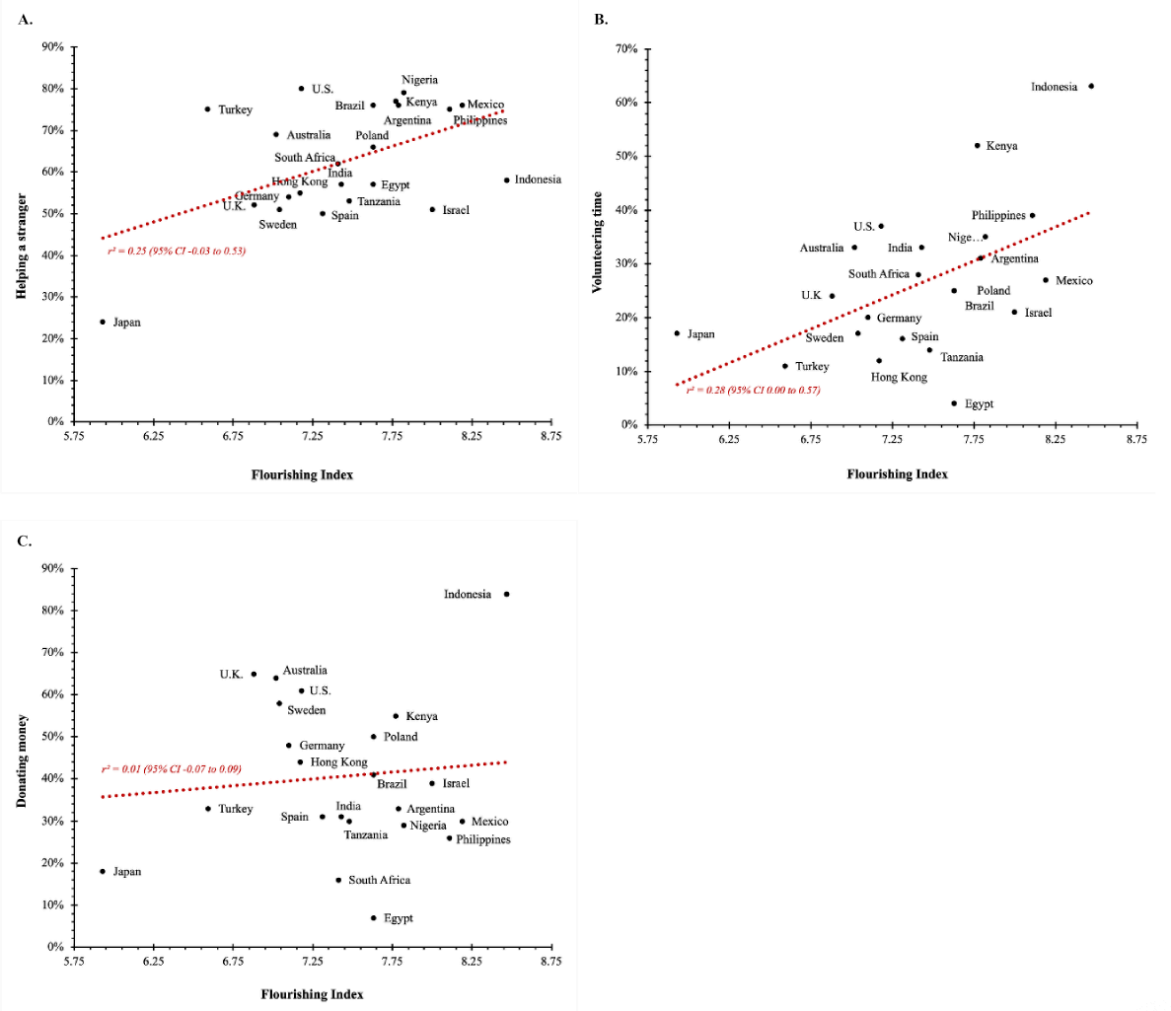
Relation between national measures of the Flourishing Index and national measures of (A) helping a stranger, (B) volunteering time, and (C) donating money. Shown for each is the linear line of best fit and the corresponding *R*^2^ (95% CI) values.

The 22 participating countries from the GFS are shown in Figure 1. There was a moderate association between the national measures of the FI and either helping a stranger (*R*^2^=0.25, 95% CI −0.03 to 0.53; Figure 1A) or volunteering time (*R*^2^=0.28, 95% CI 0.00-0.57; Figure 1B). No association was found between the FI and donating money (*R*^2^=0.01, 95% CI −0.07 to 0.09; Figure 1C).

## Discussion

The positive associations between flourishing and nonfiscal charitable behaviors, helping a stranger, and volunteering suggest that initiatives aimed at improving population well-being may encourage civic and prosocial behaviors that benefit society at large. These initiatives can drive prosocial behaviors, and thereby improve the health of both donors and recipients. Investments in public health and purpose-driven programs may indirectly increase levels of informal helping, thereby reinforcing community resilience. Conversely, the absence of any relation between flourishing and financial donations points to a need for distinct strategies to encourage monetary donations. Other studies describe a rationale for this motivational difference. For example, asking a donor for nonfiscal compensation, such as their time, can induce thoughts of well-being, whereas asking for a financial contribution tends to suppress such thoughts [5]. Others suggest that donors perceive greater control when donating their time rather than money, leading to greater participation in the former [6].

Economic factors such as national income were not assessed in the present study. Monetary forms of giving may be constrained by such factors. This study was also limited by the narrow scope of the transactional actions assessed in the WGI. Asking respondents about their participation in other forms of charitable giving could reduce measurement bias due to cultural differences. While the analysis was based on previous data, this approach is highly replicable. Future research could consider the cultural influences on charitable giving, while further discriminating between fiscal and nonfiscal ways of giving. Future studies should also consider including macroeconomic factors to identify other predictors of charitable giving.

The present findings have important implications for public health. The associations between prosocial behavior and human flourishing suggest that nonfiscal forms of charitable giving might be linked to improved cohesiveness among communities worldwide. These findings support a comprehensive view of preventive medicine, in which health promotion not only includes reducing the risk of disease but also fosters social connections and strengthens psychological well-being. Research indicates that flourishing is associated with lower all-cause mortality, suggesting that the present study may be used as a guide for initiatives aimed at promoting public health [7].

## Abbreviations

FI: Flourishing Index
GFS: Global Flourishing Study
REB: Research Ethics Board
WGI: World Giving Index
